# Comparison of minimally invasive plate osteosynthesis and conventional plate osteosynthesis for humeral shaft fracture: A meta-analysis

**DOI:** 10.1097/MD.0000000000004955

**Published:** 2016-09-30

**Authors:** Bin-feng Yu, Liang-le Liu, Guo-jing Yang, Lei Zhang, Xi-peng Lin

**Affiliations:** Department of Orthopedics, The Third Affiliated Hospital of Wenzhou Medical College, Wenzhou, Zhejiang Province, People's Republic of China.

**Keywords:** fracture, humeral shaft, meta-analysis, minimally invasive, plate

## Abstract

**Background:**

The objective of this meta-analysis was to compare the efficacy and safety of minimally invasive plate osteosynthesis (MIPO) and conventional plate osteosynthesis (CPO) for humeral shaft fracture.

**Methods:**

Potential academic articles were identified from the Cochrane Library, Medline (1966–2016.3), PubMed (1966–2016.3), Embase (1980–2016.3), and ScienceDirect (1966–2016.3). Gray studies were identified from the references of the included literature. Randomized controlled trials (RCTs) and non-RCT involving MIPO and CPO for humeral shaft fracture were included. Two independent reviewers performed independent data abstraction. *I*^2^ statistic was used to assess heterogeneity. Fixed or random effects model was used for meta-analysis.

**Results:**

Two RCTs and 3 non-RCTs met the inclusion criteria. There was a lower incidence of iatrogenic radial nerve palsy in patients with MIPO (*P* = 0.006). There was no statistically significant difference in in the risk of developing nonunion, delay union, malformation, screw loosening, infection, operation time, UCLA, and MEPS function score between the 2 groups.

**Conclusion:**

MIPO decreased incidence of iatrogenic radial nerve palsy and is an efficacy and safety technique for humeral shaft fracture. Due to the limited quality and data of the evidence currently available, more high-quality RCTs are required.

## 1 Introduction

Fractures of the humeral shaft account for approximately 1% to 5% of all adult fractures.^[1–3]^ Because nonoperative treatment of humeral shaft fracture may results in varus deformity and limitation of shoulder and elbow motion, there has been a trend toward operative treatment.^[4–6]^

Variable surgical treatment methods of humeral shaft fracture, including external fixation, open plating, and intramedullary fixation, have been reported earlier resumption of daily activity and good clinical outcomes.^[7]^ Recently, the outcomes of conventional plate osteosynthesis (CPO) have been considered to be the gold standard surgical treatment.^[8,9]^ However, open reduction and plate fixation still reveals complications such as malunion, nonunion, iatrogenic radial nerve injury, and deep infection.^[10,11]^

In theory, minimally invasive plate osteosynthesis (MIPO) preserves the enveloped soft tissue and the periosteal blood supply without directly exposing the fracture area and emphasized a biologic fixation to ameliorate fracture healing.^[12]^ Several published studies have compared MIPO with CPO in the treatment of humeral shaft fracture.^[13–17]^ Up to now, the potential benefit of MIPO has not yet been confirmed in the previous studies. Moreover, a few limitations could be observed in previous studies such as small sample, inaccurate evaluations, inconclusive results, and short-term follow-up. Therefore, we conduct a large sample meta-analysis to compare the efficacy and safety of MIPO with CPO in patients with humeral shaft fracture from randomized controlled trials (RCTs) and non-RCTs.

## 2 Methods

### 2.1 Search strategy

**Figure 1:**
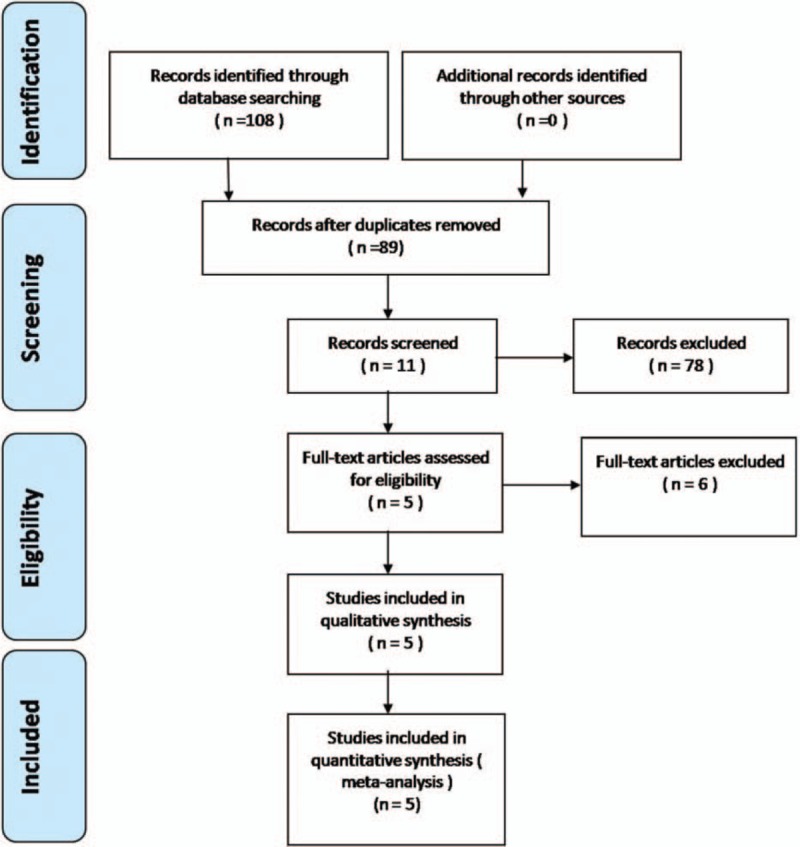
Flowchart of the study selection process.

Electronic databases including Cochrane Library, Medline (1966–2016.3), PubMed (1966–2016.3), Embase (1980–2016.3), and ScienceDirect (1985–2016.3) were searched. Gray studies were identified from the reference of included literature. No language was restricted. The search process was conducted as follows in Fig. 1. The key words “humeral shaft fracture,” “open,” “minimally invasive,” and “plate” were used in combination with the Boolean operators AND or OR. This study is a meta-analysis, which needs not the ethics committee or institutional review board to approve the study.

### 2.2 Inclusion criteria

Studies were included if the following criteria were met:

- Study design: Comparative studies (RCTs or non-RCTs).
- Population: Adult patients with humeral shaft fracture.
- Intervention group: MIPO.
- Control group: CPO.
- Outcomes measures: subjective pain perception, function score such as American Shoulder and Elbow Surgeons scale (ASES), University of California at Los Angeles scale (UCLA), Mayo elbow performance index (MEPI), Constant score, range of motion (ROM), operative time, union time, and complications.

### 2.3 Exclusive criteria

Patients were excluded from the meta-analysis if they had Gustilo–Anderson grade III open fractures, fractures extended to shoulder and elbow joints, preoperative radial nerve injury, and pathological fractures.

### 2.4 Selection criteria

For each eligible study, both reviewers extracted all the relevant data independently. Any disagreement was resolved by discussion; when no consensus could be achieved, a 3rd reviewer acted as the adjudicator and made the final decision. Contact to original authors for supplementary information was adapted when necessary.

### 2.5 Quality assessment

Two reviewers independently evaluated the bias risk of included studies. RCTs were assessed with the RCT bias risk assessment tools of the Cochrane Handbook Version 5.2.^[18]^ Non-RCTs were assessed with the Methodological Index for Non-randomized Studies (MINORS).^[19]^ Disagreements were resolved by consensus or consultation with the senior reviewer.

### 2.6 Data extraction

For each eligible study, both reviewers extracted all the relevant data independently. Any disagreement was resolved by discussion; when no consensus could be achieved, a 3rd reviewer acted as the adjudicator and made the final decision. Contact to original authors for supplementary information was adapted when necessary.

### 2.7 Data analysis and statistical methods

The meta-analysis was conducted with Review Manager software 5.1 for Windows (RevMan Version 5.1; The Nordic Cochrane Center, The Cochrane Collaboration, Copenhagen, Denmark). For continuous outcomes, the mean difference (MD) or standardized mean difference (SMD) and 95% confidence intervals (CIs) were presented. Risk difference (RD) and 95% CIs were calculated for dichotomous data. A *P* value < 0.05 was considered statistically significant. Statistical heterogeneity was assessed using a standard Chi-square test with significance set at a *P* value of 0.1, which was measured by the *I*^2^ statistic. When *I*^2^ > 50%, *P* < 0.1 was considered to be significant heterogeneity. In that case, a random-effects model was applied for data analysis. A fixed-effects model was used when no significant heterogeneity was found. In cases of significant heterogeneity, subgroup analysis was performed to investigate the sources.

## 3 Results

### 3.1 Search results

A total of 108 studies were identified as potential relevant literature reports. By scanning title and abstract, 102 reports were excluded according to the eligibility criteria. No additional studies were obtained after the reference review. Ultimately, 3 non-RCTs and 2 RCTs were eligible for data extraction and meta-analysis.^[13–17]^ The searching process is shown in Fig. 1.

### 3.2 Risk of bias assessment

**Figure 2:**
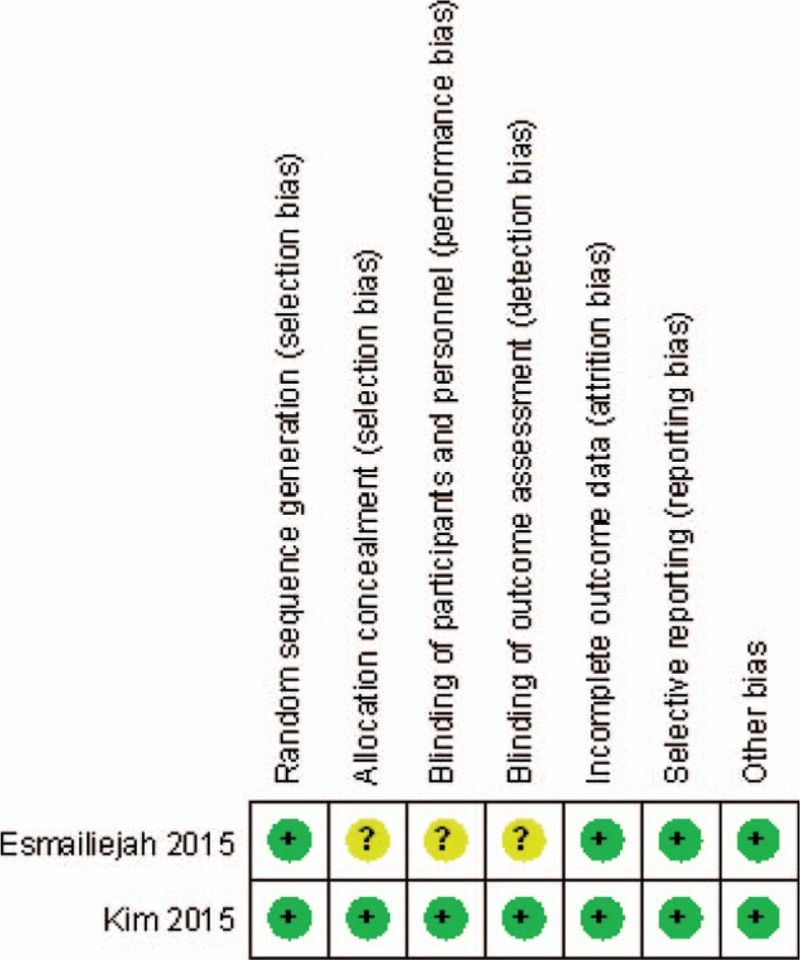
The summary of bias risk of randomized controlled trials.

The RCT quality was assessed based on the Cochrane Handbook for Systematic Review of Interventions (Fig. 2). All RCTs stated clear inclusion criteria and provided a methodology of randomization, which showed a low risk of selection bias. Adequate concealment of allocation and blind method were unclear for 1 RCT. Both 2 study performed intent-to-treatment analysis, and thus, there was a potential risk of type II statistical error. No studies showed an unclear bias due to incomplete outcome data or selective outcome reporting. For 3 non-RCTs, the MINORS score was 14 to 19 for the retrospective controlled trials. The methodological quality assessment is illustrated in Table 1 (non-RCT).

**Table 1:**
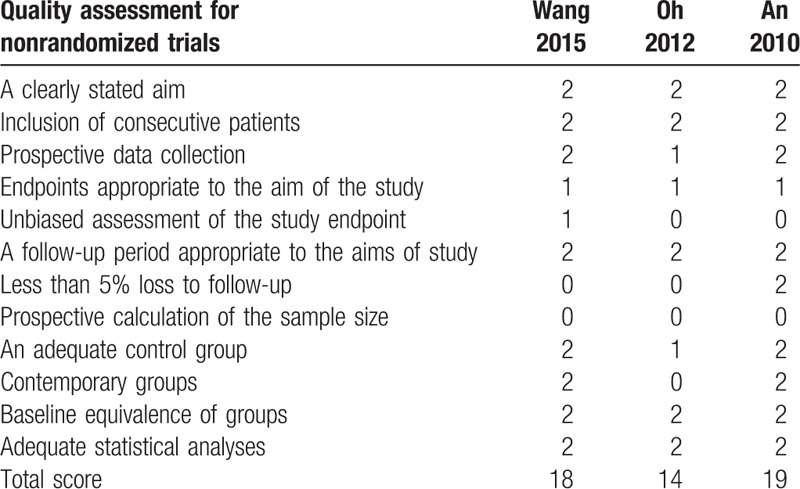
Quality assessment for nonrandomized trials.

### 3.3 Study characteristics

Demographic characteristics and details concerning the literature type of the included studies are summarized in Table 2. Statistically similar baseline characteristics were observed between both 2 groups. All studies had small sample sizes, from 33 to 68 patients.

**Table 2:**
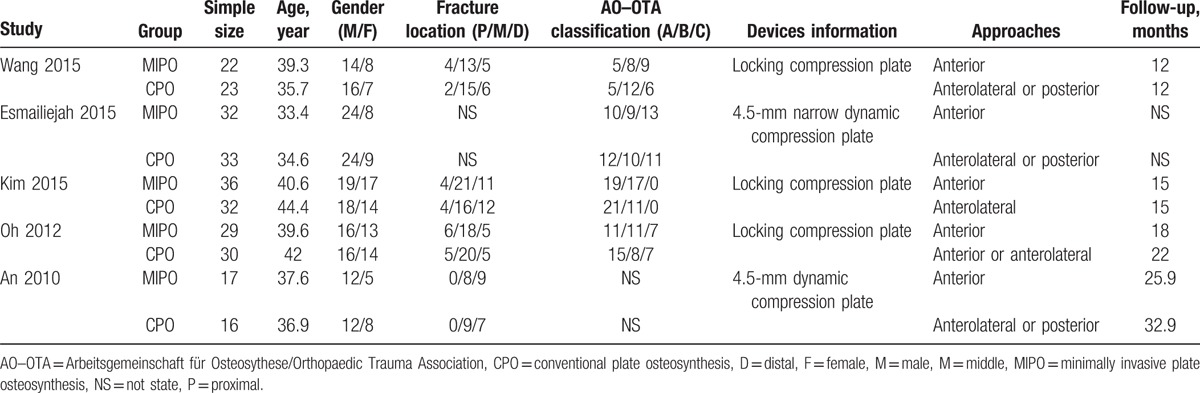
Characteristics of included studies.

### 3.4 Outcomes of meta-analysis

It was possible to perform a meta-analysis with 9 outcomes (Table 3). There were statistically significant differences between MIPO and CPO group for iatrogenic radial nerve palsy (RD = −0.08, 95% CI: −0.14 to −0.02, *P* = 0.006). With respect to other outcome, there was no statistically significant differences between MIPO and CPO groups in the risk of developing nonunion, delay union, malformation, screw loosening, infection, operation time, UCLA, or MEPS function score (Table 3).

**Table 3:**
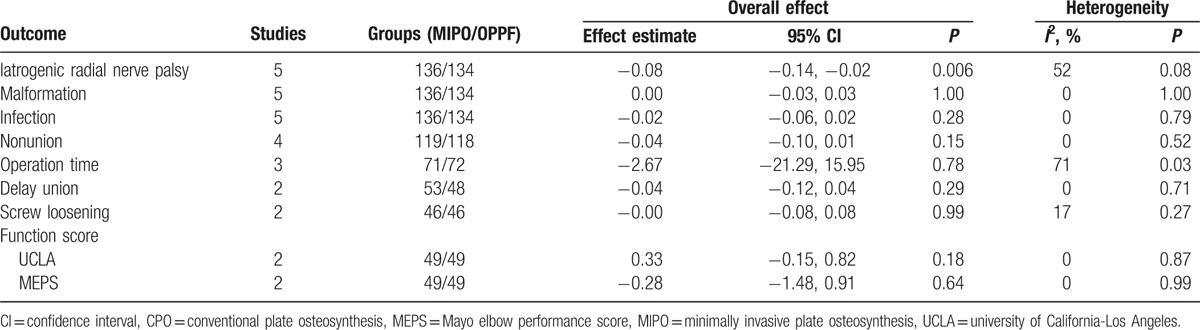
Meta-analysis results.

### 3.5 Other outcomes

Several other outcome measurements were identified, but insufficient data were provided for meta-analysis. For instance, 2 included studies An et al^[13]^ and Kim et al^[15]^ reported similar postoperative shoulder ROM between 2 groups. Wang et al^[17]^ found that significantly increased incidence of postoperative malrotation >20° was observed in the MIPO group.

## 4 Discussion

CPO could result in fracture nonunion or iatrogenic radial nerve palsy and prevent patients’ postoperative rehabilitation.^[20]^ So, a number of orthopedists have tried to find methods to solve the problem. MIPO for humeral shaft fractures was 1st proposed by Apivatthakakul et al^[21]^ through cadaveric studies. Subsequently, MIPO was adopted to avoid these complications.^[22,23]^ The most important finding of present meta-analysis is that compared with CPO, MIPO for humeral shaft fractures could decrease incidence of postoperative iatrogenic radial nerve palsy. Based on the results, MIPO is a safe technique with no significant postoperative complications for humeral shaft fractures.

Radial nerve palsy is a common complication in humeral shaft fractures and may result in extremity disability and increasing medical cost. Both ultrasound and cadaveric studies have indicated that the radial nerve is at high risk of intraoperative damage and that the procedures should be performed only by experienced surgeons.^[24,25]^ Although radial nerve exploration was routinely undertaken by most surgeons, radial nerve palsy is cited as a postoperative complication in 6.5% of conventional plate fixations for humeral shaft fractures.^[26,27]^ In our meta-analysis of the 5 included studies, pooled results indicated that MIPO contributed greatly to the prevention of postoperative iatrogenic radial nerve palsy (*P* = 0.006). Wang et al^[17]^ inferred that during the MIPO operation, the split brachialis was retracted laterally to protect the radial nerve, which may help decrease risks of intraoperative damage from surgical instruments.

The mechanism of MIPO technique is avoiding directly exposing the fracture site to preserve the enveloped soft tissue and the periosteal blood supply. Although, both incidence of nonunion and delay union in MIPO group are lower than that in CPO group, the pooled results found no significant difference in the incidence of nonunion (*P* = 0.15), delay union (*P* = 0.29), and infection (*P* = 0.28) between MIPO and CPO groups. These may be due to small sample size of included studies, including simple fractures or better protection of blood supply during CPO. Many surgeons believed that MIPO technique potentially accelerates the union process. Four included studies^[13–16]^ stated the fracture union time and the difference was not statistically significant. However, insufficient data about fracture union time were provided for present meta-analysis.

When using MIPO technique, fracture reduction was closed and indirect. Therefore, operation time is probably longer comparing with open reduction. But the pooled data in this meta-analysis found no significant differences (*P* = 0.78). All included studies reported that the surgeons were experienced at both procedures. In Oh et al^[16]^ study, the operation time was significantly longer in CPO group. As autogenous iliac bone grafting was done in 5 patients in the CPO group. On the other hand, it is inevitable to use image intensifier for MIPO to achieve a satisfactory alignment. Using image intensifier is not only require additional operation time, but also lead to intraoperative radiation exposure.^[15,16]^

Malformation is a more common complication as compared to conventional open reduction.^[28]^ In our meta-analysis, the results indicated that MIPO technique did not lead to severe malformation (*P* = 1.00) or screw loosening (*P* = 0.99). On anteroposterior and lateral radiographs, Esmailiejah et al^[14]^ and Oh et al^[16]^ both reported that there were not significant difference for angular deformity between 2 groups. Wang et al^[17]^ applied the humeral retroversion angle (HRA) by computed tomography (CT) scanning to observe the malrotation.^[17]^ Although they found that MIPO was associated with greater postoperative malrotation, this did not translate to decreased functional outcomes.

Postoperative function of shoulder is also another important element to determine the effectiveness of MIPO technique for humeral shaft fractures. Various scoring systems were used to assess functional recovery in different studies. The pooled result found similar MEPS (*P* = 0.64) and UCLA (*P* = 0.18) with no heterogeneity. Although limited data could not be extracted from 3 studies,^[15–17]^ all of included studies reported that there was no significant difference postoperative function of shoulder between 2 groups.

Several potential limitations should be acknowledged in the present meta-analysis: only 2 RCT and 3 non-RCTs were identified, and the sample sizes of the included studies were relatively small; methodological weaknesses exist in all included RCT and non-RCTs; and some data are invalid for meta-analysis, such as the postoperative ROM.

## 5 Conclusions

MIPO technique decreased incidence of postoperative iatrogenic radial nerve palsy and did not increase postoperative complications for humeral shaft fractures. More high-quality, RCTs are required for further confirming of the application of MIPO technique for humeral shaft fractures.
